# Determination of reference intervals of serum levels of human epididymis protein 4 (HE4) in Chinese women

**DOI:** 10.1186/s13048-015-0201-z

**Published:** 2015-11-09

**Authors:** Yaping Tian, Chuanxin Wang, Liming Cheng, Aimin Zhang, Wen Liu, Lin Guo, Huiming Ye, Yanchun Huang, Jing Chen, Xinyu Wen, Yuelei Xing, Guixi Zheng, Ziyong Sun, Huijun Li, Peng Zhang, Wanli Liu, Ying Chen, Zhongying Zhang, Yi Xu, Yishan Huo, Qishui Ou

**Affiliations:** Department of Clinical Biochemistry, Chinese PLA General Hospital, Beijing, 100853 China; Department of Clinical Laboratory, Qilu Hospital of Shandong University, Jinan, 250012 Shandong Province China; Laboratory Medicine Department, Tongji Hospital, Tongji Medical College of HUST, Wuhan, 430030 Hubei Province China; Department of Clinical Laboratory, Tianjin Medical University Cancer Institute & Hospital, Tianjin, 300060 China; Department of Clinical Laboratory, Sun Yat-Sen University Cancer Center, Guangzhou, 510060 Guangdong Province China; Department of Clinical Laboratory, Fudan University Shanghai Cancer Center, Shanghai, 200032 China; Department of Clinical Laboratory, Zhongshan Hospital Xiamen University, Xiamen, 361004 Fujian Province China; Clinical Laboratory Center, Tumor Hospital Affiliated to Xinjiang Medical University, Urumqi, 830011 Xinjiang Province China; Department of Laboratory Medicine, The First Affiliated Hospital of Fujian Medical University, Fuzhou, 350005 Fujian Province China

**Keywords:** Human epididymis protein 4, Epithelial ovarian cancer, Pelvic masses

## Abstract

**Background:**

To determine reference intervals for serum levels of human epididymis protein 4 (HE4) in Chinese women.

**Methods:**

In this multicenter (*n* = 9) study, 618 healthy women, 767 patients with non-malignant diseases, and 951 patients with malignant tumors were enrolled. Serum levels of HE4 were measured in all patients using electrochemiluminescence immunoassays. The influence of age, menopause, malignancy status and other characteristics on the levels of HE4 was evaluated using univariate and multivariate analyses. Confidence intervals (2.5–97.5 %) were determined in different populations.

**Results:**

There were significant differences in HE4 levels among groups with different ages, menopause or malignancy status. Higher levels of HE4 were detected in elder compared to younger, post- compare to pre- menopause and malignant compared to benign subjects. Multivariate analysis showed that menopause and malignancy status, as well as smoking and pelvic masses were independent factors involved in serum HE4 levels. In pre-menopause stage, the reference ranges of HE4 level were 29.30–68.79, 28.12–1284.83 and 34.75–981.91 pmol/L in healthy, benign and malignant populations, respectively. In post-menopause stage, the reference ranges are 35.96–114.43, 39.11–2208.70 and 39.40–1678.13 pmol/L for those populations.

**Conclusions:**

The present study has established the reference intervals of HE4 levels in pre- and post-menopause populations with different malignancy status.

**Electronic supplementary material:**

The online version of this article (doi:10.1186/s13048-015-0201-z) contains supplementary material, which is available to authorized users.

## Background

Ovarian cancer is one of the most common gynecologic cancers all over the world. The estimated annual incidence is 225,500 cases worldwide, and 140,200 patients die every year from the disease [1]. In China, the incidence of ovarian cancer has also increased in recent years, and it is now ranking the eighth most common cancer [2]. Despite the improvement of surgical techniques and development of a number of anti-tumor drugs and new therapies, the 5-year survival rate of late-stage ovarian cancer is only 30 % [3]. Therefore, early diagnosis of ovarian cancer is critical for prognosis and long-term survival. Unfortunately, due to the lack of specific symptoms at early stages, most patients are diagnosed only in late stages [4, 5].

**Fig. 1:**
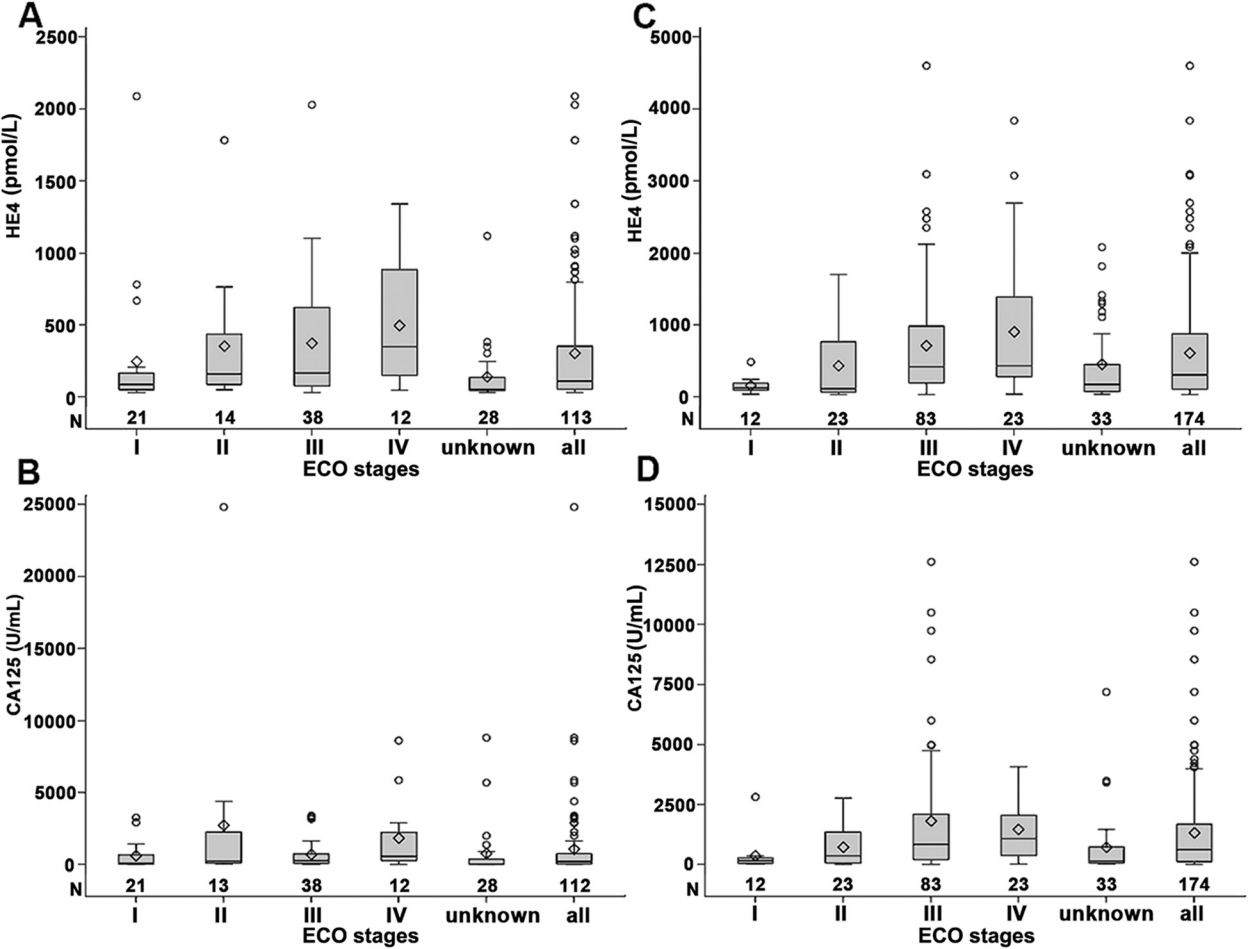
HE4 and CA125 distribution in EOC patients according to cancer stage. **a** and **c**, pre-menopause; **b** and **d**, post-menopause

The human epididymis protein 4 (HE4) (also called WFDC2) was originally identified as a small secreted protein that plays a role in sperm maturation in males [6], and it was found to be expressed in some ovarian cancers [7, 8]. Serum levels of HE4 have been shown to be useful for the diagnosis of ovarian cancer [9–11]. Not only may it be used to predict malignant status of pelvic mass, the HE4 level is also correlated with malignancy level and complexity of the disease [12]. In addition, it was shown to be correlated to surgery outcomes [13]. However, despite the existence of many methods for HE4 measurements, there is a lack of standardization and results cannot be compared between populations and studies. Previous studies have shown that age, fertility status, menopause, smoking, renal function, ethnicity, and detection method may affect serum HE4 levels [14–18]. Therefore, reference intervals of serum HE4 levels need to be established in different populations stratified according to these factors. In addition, only limited data are available on HE4 levels in Han Chinese individuals living in China [17]. Therefore, there are large variations in HE4 reference ranges, causing uncertainty in clinical application.

Nevertheless, recent studies have described HE4 as a specific and useful biomarker for early diagnosis of epithelial ovarian cancer (EOC), which account for 90 % of all ovarian cancers [19]. In addition, incorporation of serum levels of HE4 and CA125 in the Risk of Ovarian Malignancy Algorithm (ROMA) [18] for clinical evaluation have resulted in improved EOC diagnosis specificity and sensitivity, and have helped distinguish malignant from benign pelvic masses [20, 21]. Other studies have equally demonstrated the importance of HE4 in predicting ovarian cancer recurrence [22, 23].

Therefore, the present multicenter study was undertaken to determine the factors influencing HE4 levels and the reference intervals of HE4 levels in a Chinese population.

## Methods

### Study participants

This multi-center (*n* = 9) study prospectively included healthy women and female patients (*n* = 2351) from October 2012 to February 2013. All participating sites received the approval of their ethics committee. The need for informed consent was waived by the committees since all specimens used in the present study were leftover samples.

The inclusion criteria for all individuals were: 1) Clinical records including age, menopause status, race, sex, smoking history, diagnosis of benign disease patients and pathological results were available; and 2) normal appearance of blood samples, with at least 0.5 ml being available. The exclusion criteria were: 1) patients <18 years old; 2) incomplete clinical data; 3) insufficient blood sample volumes (<0.5 ml); 4) blood stored or shipped at >0 °C; 5) samples submitted to >3 freeze/thaw cycles; or 6) blood sample with icteric, lipemic, hemolytic appearance or particles. Clinical diagnosis of the subjects included apparently healthy (618 specimens), non-malignant diseases (767 specimens) and malignant tumors (951 specimens including 287 EOC).

EOC diagnosis was confirmed by pathological analyses, and the EOC surgical staging was recorded.

### HE4, CA125, E2, and Prog

All blood samples were taken at the time of primary diagnosis. At the time of blood sampling, no patient has been experienced any chemo- or radio- therapy. Blood specimens were centrifuged to obtain serum (at least 0.5 ml), and samples were frozen at −80 °C until analysis. HE4 and CA125 serum levels were determined in all samples using a Roche Elecsys Cobas 601 platform (Roche Diagnostics, USA) using specific assays and kits from for the detection of HE4 (Roche Diagnostics, Basel, Switzerland) according to the manufacturer’s instructions. The assay method used was electrochemiluminescence immunoassay (ECLIA) and the measurement range was 15.0–1500 pmol/L for HE4 All laboratories involved in this study passed the External Quality Assessment (EQA)/ISO 15189, as required by Chinese authorities. The same batch number reagents and controls were used in laboratories in all sites. All these laboratories passed the external quality assessment before the study.

### Statistical analysis

Statistical analyses were performed using SPSS 18.0 (IBM, Armonk, NY, USA). Box-Cox regression was used for data transformation to obtain a normal distribution. Quantitative values are reported as N, median (range). Qualitative variables were described as numbers. One way ANOVA and Bonferroni post hoc test were used for comparisons. Reference intervals were determined by selecting the 2.5 and 97.5 percentiles. Multivariate linear regression analysis was used to evaluate correlations between different factors with HE4 levels. Differences were considered statistically significant at *P* < 0.05.

## Results

### Study population

A total of 2351 subjects were enrolled in the study including 618 healthy volunteers, 287 patients with epithelial ovarian cancer according to Federation International of Gynecology and Obstetrics (FIGO) staging and 1446 patients with various benign diseases and other cancers. The medium age was 49 (range 18–95) years. There were 1213 subjects in pre-menopause, 1137 post-menopause and 1 with unknown status (Table 1).

**Table 1:** Characteristics of the study population

Parameter	*n* (%)
Menopausal	
Pre-menopause	1213 (51.6)
Post-menopause	1137 (48.4)
Unknown	1
Healthy	618 (26.3)
Benign disease	767 (32.6)
Benign gynecologic tumor	348
Other gynecological disease	70
Pregnant	108
Congestive heart failure	43
Renal failure	74
Other benign diseases	124
Malignant disease	951 (40.5)
Epithelial ovarian cancer (EOC)	287
Stage I	33
Stage II	37
Stage III	121
Stage IV	35
Unknown	61
Endometrial cancer	136
Breast cancer	178
Gastrointestinal cancer	139
Lung cancer	145
Bladder cancer	66
Mixed benign and malignant disease	15 (0.6)
Total	2351
Age median (range)	49 (18–95)

In the analysis of HE4 levels, 15 samples with combined benign and malignant diseases, 1 sample with unknown menopause status, and 4 samples with unspecified out-of-upper-limit HE4 levels were excluded, resulting in 2331 samples included in the final analysis. The numbers of samples included and excluded in different sitess were listed in Additional file 1: Table S1. To gain a rough estimate of HE4 levels in the general population, the reference ranges (95 % CI) of HE4 levels in the healthy population in this study were evaluated in different age groups as well as in pre- and post- menopause subjects (Table 2).

**Table 2:** HE4 reference range in healthy populations according to age or menopause status

	Number	Value (mean ± SD)	Reference range (2.5–97.5 %)
Ages*			
<40	201	45.85 ± 10.72	29.25–68.50
40–49	102	47.80 ± 9.73	32.11–68.96
50–59	104	54,11 ± 14.30	33.04–88.67
60–69	108	59.91 ± 14.33	34.72–92.35
≥70	103	77.57 ± 23.20	45.18–132.00
Pre-menopause	308	46.61 ± 10.70	29.25–68.96
Post-menopause	310	63.94 ± 20.21	34.72–114.90
Overall	618	55.30 ± 18.35	31.82–105.10

*A statistical significant difference in HE4 was found between each age group (*p* < 0.0001) except between <40 and 40–49

### Factors that influence HE4 levels

It has been previously noticed that HE4 level in individuals could be affected by various factors including age, menopause and diseases [14–18]. To determine which factors could interfere with HE4 level, study subjects were grouped based on age, menopause, and disease. As shown in Table 3, significant differences in HE4 levels were detected in patients grouped in each category. Aging, menopause, malignant diseases and increased staging of ovarian cancer seemed all result in increased levels of HE4. These results indicate that multiple interfering factors have to be considered to establish a reference interval of HE4 level.

**Table 3:** Influences of age, menopause or malignancy on HE levels

Parameter	Number	HE4 Value	*P*
		Median (range)	
Age			<0.001
<40	628	47.275 (15–2982)	
40–49	544	51.01 (24.07–4322)	
50–59	520	59.47 (26.08–5234)	
60–69	371	66.63 (23.3–3844)	
≥70	268	84.33 (24.22–2276)	
Menopause			<0.001
Pre-menopause	1206	48.81 (15–4322)	
Post-menopause	1125	68.46 (23.3–5234)	
Malignancy status			<0.001
Healthy	618	51.09 (23.3–166)	
Benign	765	51.92 (15–5234)	
Malignant	948	67.935 (26.08–4603)	
Overall	2331	56.51 (15–5234)	

To confirm the interfering factors of HE4 levels in individual, multivariate linear regression analyses were used to identify the correlations between different characteristics and HE4 levels. As shown in Table 4, when only age, menopause and malignancy status were considered, menopause and malignancy status, but not age, were independently associated with HE4 levels. When pregnancy status, smoking, and existence pelvic masses were added into the analysis, menopause and malignancy status were still independently associated with HE4 levels. Smoking and pelvic masses were also significantly associated with HE4 levels. Age seemed not to be an independently associated factor.

**Table 4:** Multivariate analysis of independent correlated factors of HE4 level

Parameter	Coefficient	*P*
Model 1		
Age	0.691	0.432
Menopause	65.889	0.014
Malignancy status	64.385	<0.001
Model 2		
Menopause	90.199	0.001
Smoking	212.286	0.006
Pelvic masses	68.517	<0.001
Malignancy status	53.140	<0.001

Model 1: age, menopause and malignancy status were included. Model 2: age, menopause, malignancy status, pregnancy, smoking, and pelvic masses were included. The associations with HE4 levels were analyzed using multivariate linear analysis using the enter regression method

### HE4 reference range in different populations

Results indicated that the presence of an ovarian tumor and menopause status, but not age, had to be considered when determining whether HE4 levels are abnormal. The post-menopausal population generally had higher HE4 levels compared with pre-menopausal individuals. The presence of a malignant tumor, mostly ovarian cancer, could further increase HE4 levels. The detailed reference intervals of HE4 levels in healthy, individuals with benign tumors or malignant tumors, for pre- and post-menopause populations, are listed in Table 5. In pre-menopause stage, the reference ranges of HE4 level were 29.30–68.79, 28.12–1284.83 and 34.75–981.91 pmol/L in healthy, benign and malignant populations, respectively. In post-menopause stage, the reference ranges are 35.96–114.43, 39.11–2208.70 and 39.40–1678.13 pmol/L for those populations. In addition, HE4 and CA125 distribution in patients of different EOC stages also showed significant differences (Fig. 1).

**Table 5:** Reference ranges of HE4 in different populations

Populations		Reference range of HE4
Pre-menopause	Healthy	29.30–68.79
	Benign	28.12–1284.83
	Malignant	34.75–981.91
Post-menopause	Healthy	35.96–114.43
	Benign	39.11–2208.70
	Malignant	39.40–1678.13

## Discussion

As mentioned previously, ovarian cancer at early stages can be effectively treated with surgery with significantly higher 5-year survival rate [3]. However, most ovarian cancers are diagnosed in their late stages and this is also one of the reasons why the acceptance of laparoscopic surgeries for ovarian cancer is much slower than that for other malignant female reproductive cancers [24]. Ghezzi et al. [25] and Bae et al. [26] described that minimally invasive surgeries, such as laparoscopic staging or restaging of early stage ovarian cancer are as safe and effective as open abdominal surgery. A systemic review also demonstrated that patients undergoing laparoscopic surgeries had lower blood loss, shorter hospital stay period and shorter time interval between surgery and chemotherapy compared with open abdominal surgery [27]. Therefore, diagnosis of the disease in early stages plays a critical role in the timely treatment and will bring more benefits for the patients. As a correlated biomarker with ovarian disease, HE4 may facilitate to optimize the early diagnosis of ovarian cancer. To the best of our knowledge, this is the first prospective multicenter study examining age-related reference ranges of serum HE4 levels in a Chinese population. The 2.5–97.5 % cut-offs were determined in different patient subpopulation. There were significant differences in HE4 levels among groups with different ages, menopause or malignancy status. Multivariate analysis showed that menopause and malignancy status, as well as smoking and pelvic masses were independent factors involved in serum HE4 levels.

Different studies indicated different effects of patients’ characteristics on HE4 levels. Ferraro et al. [14] have shown that the menopausal status was associated with HE4 levels, as well as smoking and renal function. Speeckaert et al. [11] have shown that menopause was associated with HE4 levels. A meta-analysis supported the previous point of view and also showed that the detection method was associated with HE4 variations as well [16]. A study has shown that longitudinal measurement of HE4 levels might be better than cross-sectional ones [28]. but the applicability of this approach in the clinical setting might be more difficult. A study in multiple Asian ethnicities revealed that age was associated with HE4 levels, as well as ethnicity (Malays vs. Indians and Chinese) [17]. Moore et al. [15] have shown that age, menopausal status and pregnancy were associated with HE4 levels. Taken together, these studies suggest that there are variations in the factors influencing HE4 levels among different populations. These differences might be population-specific, or may be the result of the small sample size observed in some studies. Further studies focusing on specific populations should determine the factors influencing HE4 levels before generalizing to other populations. In the present study, menopause, malignancy status, smoking, and pelvic masses were independent factors associated with serum HE4 levels.

A recent meta-analysis revealed that the capacity of HE4 in identifying malignant from benign lesions was high [16]. Beside the cancer itself, the histological type and cancer stage are associated with HE4 levels as well [11]. HE4 levels in EOC patients in this study were significantly higher than the subjects with benign diseases. Apparently the diagnostic value of HE4 level would be only significant at the early disease stages. Our data showed that the HE4 levels in stage I/II patients were significantly higher than that in subjects with benign ovarian disease, further indicating the value of HE4 as an early diagnosis marker. A study has shown that HE4 levels were better than CA125 levels for detecting ovarian cancer [29], which is supported by a meta-analysis [30]. A recent study revealed that the combination of HE4 levels with pelvis ultrasound achieved the best sensitivity for detecting ovarian cancers among different algorithms tested [31]. Further study is necessary to determine if the combination of HE4 measurements with other diagnostic modalities could promote the detection of ovarian cancer. Algorithms could be designed for the rapid screening of asymptomatic women.

The present study suggested that while using HE4 level as a potential indicator of ovarian cancer, other factors for each individual such as menopause, but not age, must be considered. Reference ranges need to be established after considering those interfering factors. The strength of the present study is that it had a relatively large sample size and samples were from multiple centers. However, there was also a limitation due to the variety of interfering factors, and the unavailability of some factors in a retrospective setting. Further study with larger sample size is needed to establish reliable reference ranges.

## Conclusion

In conclusion, the present study has established the reference intervals of HE4 levels in pre- and post- menopause populations with different malignancy status.

## Additional file

Additional file 1: Table S1. The detailed numbers of subjects from different centers. (DOCX 17.3 kb)

## Abbreviations

HE4: Human epididymis protein 4
EOC: Epithelial ovarian cancer
ROMA: Risk of ovarian malignancy algorithm
ECLIA: Electrochemiluminescence immunoassay
EQA: External quality assessment
ECLIA: Electrochemiluminescence immunoassay
