# Supplementary material for: Determination of reference intervals of serum levels of human epididymis protein 4 (HE4) in Chinese women
Source: J Ovarian Res. 2015 Nov 9;8:72. doi: 10.1186/s13048-015-0201-z (PMC4637994; doi:10.1186/s13048-015-0201-z)
Supplement: Additional file 1: Table S1. — The detailed numbers of subjects from different centers. (DOCX 17.3 kb) [file 13048_2015_201_MOESM1_ESM.docx]

**Supplemental table 1.** The detailed numbers of subjects from different centers.

| **No.** | **Site** | **Screened** | **Excluded** |
| --- | --- | --- | --- |
| 1 | Chinese PLA General Hospital, Beijing | 161 |  |
| 2 | Qilu Hospital of Shandong University, Jinan | 414 | 1 |
| 3 | Tongji Hospital, Tongji Medical College of HUST, Wuhan | 368 | 1 |
| 4 | Tianjin Medical University Cancer Institute & Hospital, Tianjin | 313 | 1 |
| 5 | Sun Yat-Sen University Cancer Center, Guangzhou | 262 | 1 |
| 6 | Fudan University Shanghai Cancer Center, Shanghai | 297 |  |
| 7 | Zhongshan Hospital Xiamen University, Xiamen | 191 | 1 |
| 8 | The Affiliated Tumor Hospital of Xinjiang Medical University, Urumqi | 173 | 12 |
| 9 | The First Affiliated Hospital of Fujian Medical University, Fuzhou | 172 | 3 |
|  | Total | **2351** | **20** |
